# Fatty Acid Prof iles of Ram's Sperm after Removing Some Fatty Acid Sources from the Diets and Persistency of Fatty Acids in Sperm

**Published:** 2012-03-20

**Authors:** Vahid Esmaeili, Abdolhossein Shahverdi, Ali Reza Alizadeh, Hiva Alipour, Armin Towhidi, Morteza Zarrabi

**Affiliations:** 1 Department of Embryology, Reproductive Biomedicine Research Center, Royan Institute for Reproductive Biomedi- cine, ACECR, Tehran, Iran; 2 Department of Animal Science, Saveh Branch, Islamic Azad University, Saveh, Iran; 3 Department of Animal Science, Faculty of Agricultural Science and Engineering, University of Tehran, Karaj, Iran; 4 Department of Andrology, Reproductive Biomedicine Research Center, Royan Institute for Reproductive Biomedicine, ACECR, Tehran, Iran

**Keywords:** Fatty Acids, Ovine, Spermatozoa

## Abstract

**Background:**

Mammalian spermatozoa are characterized by a high proportion of polyunsaturated fatty acids (PUFAs), but reliable data concerning dietary effects on fatty acid (FA) profile in ram's sperm and the persistency of FA in the ration to the FA in sperm has not been reported. Therefore, the aim of this study was to determine the stability of saturated and unsaturated FAs in ram's sperm despite removing FA sources from their diet.

**Materials and Methods:**

Nine Kalkoohi rams were used in a completely randomized design and they were assigned to 3 groups. The treatments were diet supplemented (35 g/d/ram) by C16:0 (RP-10®), C18: 2 (Sunflower oil; SO) and n-3 (Fish oil; FO) with Vitamin E. Fifteen weeks after the start of the supplemented diet, rams were offered a basal diet without any supplementary FA source for 35 days when the sperm’s FA ratio was determined. The data were analyzed by ANOVA (Analysis of variance) using the General Linear Model (GLM) procedure of SAS Institute.

**Results:**

Thirty five days after removing the fat supplement from the diet, major FA in sperm consisted of: C14:0, C16:0, C18:0, C18:1 cis, C18:2 cis and C22:6 n-3 docosahexaenoic acid (DHA). The percentage of C14:0 (p=0.8) and C18:1 cis (P =0.4) were similar among all the treatments. Interestingly, 35 days after the removal of fatty acid source, the percentage of C22:6 was highest in the FO treated group.

**Conclusion:**

The different sperm FA profile among various groups suggests that dietary FA had significant direct or indirect impacts on sperm FA profile after 35 days which might lead to physical and chemical changes in sperm characteristics.

## Introduction

The membrane structure of spermatozoa plays a crucial role in fertilization. The lipids of the spermatozoa have been suggested to be important for the viability, maturity, and functions of spermatozoa. It has also been suggested that the proportion of unsaturated fatty acids (UFA) may have an influence over the physical properties of the sperm membrane including membrane fluidity (1, 2). Phospholipids (PL) of spermatic cells have very high proportions of long chain polyunsaturated fatty acids (LCPUFA), especially from the n-3 and n-6 series. In many mammalian species, up to 60% of the total fatty acids are LCPUFA of the n-3 series (3, 4). The sperm of most mammalian species such as bull, monkey, human and ram show very high levels of C22: 6n-3 docosahexaenoic acid (DHA) (5). Mammals are unable to synthesize C18:2 and C18:3 fatty acids (FAs) even though they are major precursor for another long chain FAs, thus, they are recognized as essential fatty acids and need to be provided through the diet (6). Moreover, Conquer et al. (7) have concluded that using DHA in the human diet causes increasing of DHA concentration in sperm and furthermore it appears that dietary FAs transfer to sperm.

As a basic inception in ruminant, biohydrogenation (BH) of PUFA is a part of lipid digestion in the rumen and lipids are extensively altered in the rumen, resulting in marked differences between the fatty acid profile of lipids in the diet (mostly UFA) and lipids leaving the rumen (mostly saturated fatty acid). It is surprising to note that some fatty acids can escape from this process and reach the small intestine and can be found in some organs such as the mammary gland or testis. Also, they may stimulate some genes which participate in FAs metabolism in the organs (8).

Digestion, absorption, transport and metabolism of fatty acids in the body, could be affected by the changes in the structure of fatty acids leaving the rumen, or the cis to trans configuration switch (9). The main important intracellular substrate for respiration activity in mammalian spermatozoa, are phospholipids, mainly the plasmalogen fraction (10).

In particular, ram spermatozoa are able to oxidize C14:0 and C16:0 bound to plasmalogen for energy production, in this regard C16:0 and C18:1 has been reported to exist at higher levels in ram sperm (11). FAs in the diet are known to affect fatty acid composition of the tissues in all animal species. Composition of fat source in the diet can change the sperm fatty acid’s arrangement (12, 13).

Essential fatty acid (EFA) concentration can be altered by any change in the ratio of UFA n-3 and n-6. A recent report confirmed that dietary fish oil (FO) increased the proportion of DHA (C22:6 n-3) in sperm fatty acid composition (13). Since each FA influences other fatty acids functions, fat composition in diet plays an important role in the metabolism of polyunsaturated fatty acid (PUFA) in body tissues (12).

To our knowledge, no studies to date have focused on sperm FAs persistency or compared the effect of feeding palm with unsaturated fatty acid sources on the ram sperm FAs. Therefore, the objective of this study was to determine the persistence of saturated and unsaturated dietary FAs in ram sperm FA profiles 35 days after removing the FA sources from the diet.

## Materials and Methods

All chemical reagents were obtained from Sigma (St. Louis, MO, USA) unless otherwise indicated. This study received the approval of the Ethics Committee of Royan Institute.

### Experimental location

This study was carried out from September to December 2009, in the research farm of Saveh University located in Saveh district in central Iran at Latitude: 50° 21´ N, Longitude: 35° 2´ E and an Altitude of 995 m above sea level.

### Experimental animals, feeding and design

Nine Kalkoohi rams (aged 32 ± 5 months and 65 ± 5 kg average body weights) were used in a completely randomized design and they were assigned to 3 groups (n=3) and different pens in separate groups according to the type of supplementation. Rams were allocated to the treatment groups based on principal component analysis of sperm quality, prior to commencement of the trial in order to balance both high and low quality semen producing rams between treatments.

Experimental groups were offered an isoenergetic and isonitrogenous ration [metabolizable energy (ME) was 2.95 Mcal/kg in dry matter (DM) and crude protein (CP) was 11% in dry matter], which were given in two equal quantities per day and rams were allowed to walk freely. Diet was formulated to meet the nutrient requirements of AFRC (1995) for rams (14). The treatments were basic diet, supplemented with Palmitic acid (C16:0 FA) [from RP-10®; RP-10 Fat powder, Energizer Co., Johor, Malaysia]; Linoleic acid (C18:2 FA n-6) [from sunflower oil (SO); Nina sunflower oil, FRICO Co., Tehran, Iran] and n-3 FA [from Kilka fish oil (FO); Khazar fish powder Co., Kia-shahr port, Iran]. Fatty acid profile of fat sources was analyzed by gas chromatography (GC) in Oilseed Research & Development Co. lab (Tehran, Iran). All of the groups adapted to oil/fat consumption within 3 weeks and after this period each ram could feed 35 g/d of each fat source. Diet ingredients per day for all of the groups consisted of: alfalfa hay (50%), barley grain (23%), wheat straw (19%), beet molasses (5%) and vitamin E supplement (0.5%) of dietdry matter. Water and mineral blocks were available ad libitum.

After the trail period, all groups were offered basal diet without any supplementary FA sources for 35 days.

### Semen collection and lipid of sperm analysis

Semen samples from each ram were collected on the last day of feeding on the FA supplemented diet using an artificial vagina (AV). A second semen sample was collected 36 days after excluding fat sources from the diet. Semen from individual rams was transferred from the farm to the laboratory in a portable incubator at 37°C (K-systems G95, Denmark).

The semen was washed twice using an equal volume of Dulbecco’s Phosphate Buffered Saline solution (DPBS) (Sigma Aldrich Co.) followed by vortexing for 10 minutes and centrifugation (Hettich, EBA 20, Germany) for 20 minutes at 700 × g. Total lipids were extracted from spermatozoa after homogenization in a suitable excess of chloroform: methanol (2:1, v:v) (15) and trans-methylated by a solvent system of methanol: hexane (2:1, v:v) (16). The resultant fatty acid methyl esters were analyzed by GC performed with an Agilent GC 6890 system (Agilent Technologies Co.USA) coupled with a hydrogen flame ionization detector (FID) equipped with a capillary column system (BPX-70: 120 m × 0.25 mm, 250 μm i.d, 0.2 μm film thickness, Agilent Technologies).

### Statistical analysis

The data generated from the study were analyzed by ANOVA (Analysis of variance) using the General Linear Model (GLM) procedure of SAS Institute (1999) (17). Differences between treatment means were tested by Duncan’s Multiple Range Test.

## Results

FA composition of fat sources in the diet was analyzed by GC and has been shown in table 1. At the end of the experiment (35 days after excluding fatty acids from the diet) major sperm fatty acids of the experimental rams consisted of: C14:0, C16:0, C18:0, C18:1 cis, C18:2 cis and C22:6 (DHA) (Tables2, 3).

**Table 1:** Fatty acid profile of fat sources in experimental diets


Fatty acids composition (%)	RP10®^1^	SO^2^ (n-6)	FO^3^ (n-3)

**C12:0**	0.4	-----	4.03
**C14:0**	3.8	0.09	1.1
**‍C15:0**	0.2	-----	0.2
**C16:0**	89	8.5	21.2
**C16:1**	0.14	0.1	6.6
**C17:0**	0.06	0.04	1.5
**‍C17:1**	0.08	0.03	1.03
**C18:0**	0.78	4.2	5.9
**C18:1t^4^ n-9**	0.05	0.05	0.3
**C18:1c^5^ n-9**	4.3	25.6	34.1
**C18:2t n-6**	0.03	0.4	0.2
**C18:2c n-6**	0.13	59.8	2
**C18:3 gamma**	-----	-----	0.12
**C18:3 alpha n-3**	-----	0.2	1.6
**C20:0**	-----	0.3	0.3
**C20:1**	-----	0.15	2.4
**C21:1**	-----	-----	0.3
**C22:0**	-----	0.5	0.7
**C22:1**	-----	-----	0.5
**C20:5 n-3**	-----	-----	5.8
**C24:0**	-----	-----	0.3
**C24:1**	-----	-----	0.8
**C22:6 n-3**	-----	-----	8.8


1: RP10®: Provided by Sana Dam Pars Co. (Tehran, Iran), 2: SO: sunflower oil, 3: FO: fish oil, 4: trans, 5: cis.

The concentration of C18:0 in both FO (15.5 %) and SO (14.3 %) treatment groups were higher compared to RP10 (10 %) in samples collected after feeding on FA supplemented diet and basal diet.

DHA concentration increased dramatically with FO at the end of the study (20.3 %) compared to other treatments.

35 days after removing of FA sources C14:0 (p=0.8) and C18:1 cis (p=0.4) showed a similar percentage among the treatments.

The C16:0 percentage was decreased significantly (p < 0.01) as FO was added to the diet (26.18, 27.03 vs. 20.35 % of total FA in RP-10, SO and FO, respectively). The FO treatment had the lowest C16:0 percentage (p < 0.01) along all treatment groups. The C18:0 percentage was significantly different in RP-10 compared with other groups (10 vs. 14.3 and 15.5 % of total sperm FA in RP-10, SO and FO, respectively; p < 0.01).

**Table 2:** Fatty acids composition of sperm at the end of the fat supplemented diet feeding (after 12 weeks) (Mean ± SD)


Fatty acid (% of total fatty acid)	Treatments			P value^4^
	RP10®^1^	SO^2^	FO^3^		

**C14:0**	6.3 ± 0.31^b^	8.3 ± 0.2^b^	13.4 ± 1.4^a^	**
**C16:0**	25.5 ± 2.2	28.3 ± 2.9	28 ± 1.6	0.12
**C16:1**	0.22 ± 0.07^b^	0.4 ± .15^a^	0.28 ± 0.06^b^	**
**C17:0**	0.21 ± 0.5^b^	0.5 ± 0.01^a^	0.3 ± 0.05^b^	*
**C18:0**	7.4 ± 0.8 b	14.8 ± 0.4^a^	14.5 ± 0.4^a^	**
**C18:1 cis n-9**	30.7 ± 1.9^a^	23.5 ± 3^b^	10.7 ± 1.4^c^	**
**C18:2 cis n-6**	15.2 ± 1.45^a^	10.2 ± 0.43^b^	4.2 ± 0.15^c^	**
**C20:0**	0.44±0.1	0.38 ± 0.2	0.34 ± 0.17	0.8
**C20:1**	0.29 ± 0.03^a^	0.2 ± 0.02^b^	0.12 ± 0.02^c^	**
**C22:0**	1.4 ± 0.91^b^	2.1 ± 0.63^b^	3.4 ± 0.09^a^	**
**C20:3 n-6**	0.21 ± 0.3^b^	0.9 ± 0.19^a^	0.42 ± 0.28^b^	*
**C24:0**	0.1 ± 0.04^b^	0.5 ± 0.07^a^	0.11 ± 0.02^b^	*
**C24:1**	0.07 ± 0.05^b^	0.4 ± 0.22^b^	2.3 ± 0.15^a^	*
**C22:6 n-3**	9.9 ± 0.2^b^	8.7 ± 0.33^c^	20.3 ± 0.95^a^	**


SD: Standard deviation, 1: RP10®: Provided by Sana Dam Pars Co. (Tehran, Iran), 2: SO: sunflower oil, 3: FO: fish oil, 4: a, b, c: values with differing letters within the same rows are significantly different (*p<0.05; **p<0.01).

**Table 3:** Fatty acids composition of sperm lipids measured in semen samples 35 days after excluding FA sources from the diet (Mean ± SD)


Fatty acid (% of total fatty acid)	Treatments			P value^4^
	RP10®^1^	SO^2^	FO^3^		

**C14:0**	10.18 ± 0.5	9.98 ± 0.7	11.32 ± 1.1	0.8
**C16:0**	26.18 ± 1.3^a^	27.03 ± 0.9^a^	20.35 ± 1.8^b^	**
**C16:1**	0.28 ± 0.9	0.3 ± 0.03	0.39 ± 0.18	0.2
**C17:0**	0.58 ± 0.06^b^	0.71 ± 0.09^a^	0.28 ± 0.04^c^	*
**C18:0**	10 ± 0.58 ^b^	14.3 ± 1.2^a^	15.5 ± 1.3^a^	**
**C18:1 cis n-9**	27.7 ± 3.1	23.3 ± 1.7	21.6 ± 2.5	0.14
**C18:2 cis n-6**	11.9 ± 1.6^a^	9.6 ± 1.9^a^^b^	7.7 ± 2^a^	**
**C20:0**	0.48 ± 0.04	0.49 ± 0.04	0.5 ± 0.03	0.8
**C20:1**	0.33 ± 0.1	0.47 ± 0.22	0.25 ± 0.1	0.5
**C22:0**	1.01 ± 0.8^b^	1.26 ± 0.62^b^	2.36 ± 0.2^a^	**
**C20:3 n-6**	0.36 ± 0.04^b^	1.45 ± 0.34^a^	0.58 ± 0.08^b^	*
**C24:0**	0.2 ± 0.09	0.32 ± 0.06	0.47 ± 0.08	0.3
**C24:1**	0.17 ± 0.1^b^	0.6 ± 0.44^b^	2.75 ± 0.9^a^	**
**C22:6 n-3**	8.4 ± 0.6^b^	8.9 ± 0.82^b^	13.6 ± 1.6^a^	**


SD: Standard deviation, 1: RP10®: Provided by Sana Dam Pars Co. (Tehran, Iran), 2: SO: sunflower oil, 3: FO: fish oil, 4: a, b, c: values with differing letters within the same rows are significantly different (*p<0.05; **p<0.01).

The C24:1 percentage had the highest level in FO group after feeding on FA supplemented diet and 35 days after removal of FA sources.

Interestingly, C22:6 percentage was highest in FO treatment (8.5, 8.9 vs. 13.6 % of total sperm FA in RP-10, SO and FO, respectively; p < 0.01) 35 days after excluding supplementary fat source.

## Discussion

Previous studies observed a decrease in C18:1 in response to feeding of FO, marine algae, or fish meal in ruminant products such as milk. This decrease of C18:1 is a typical response to FO supplementation which appears to be a result of ruminal biohydrogenation and release of FAs to ruminant products (9, 12).

As expected, DHA concentration increased dramatically with FO at the end of this study which supports Samadian et al. findings (13) which assessed the effects of FO in ram diet.

Our results also conform the results of Salem et al. (18) which reported an increased in the amount of 22:6n-3 in the rat spermatozoa after replacing commercial rat feeding with DHA-enriched nutriceutical.

It seems that the use of PUFA sources can have positive effects on C18:0 percentages due to the biohydrogenation of PUFA in ruminants which leads to the production of C18:0.

In boars, changes in fatty acid proportions of sperm phospholipids and improvement in sperm quality appeared only after 5 weeks of feeding them marine oil. It was noteworthy that spermatogenesis and epididymal transport required 34 and 10 days respectively in boars, whereas the corresponding intervals were approximately 49 and 9 days in rams (13, 19).

It was therefore surprising to find that diet FAs changed the sperm fatty acids profile after 35 days, because sperm FA patterns in previous studies were not measured after removing FAs from the diet.

Such a modification might have accounted for the aforementioned changes in the sperm FA profile. The vast majority of research on the supplementation of unsaturated fatty acids for improved sperm production or cryosurvival has resulted in positive or at least neutral findings, with improvements noted in roosters (20), turkeys (21), boars (22), stallions (23), rabbit (24) and ram (13) fed on diets high in n-3 LCPUFA (usually C22:6).

In 1961, Hartree and Mann (11) reported higher levels of C16:0 and C18:1 in ram sperm which conforms to the results of the palm group. Hall et al. (25) reported that biomembrane fluidity and the degree of PUFA unsaturation increases when spermatozoa pass from the caput to the cauda of the epididymis. In rats it has been shown that both long-chain n-6 and n-3 PUFA can be synthesized both in germinal and Sertoli cells under hormonal control (26), indicating an active sperm lipid metabolism of the testis including the desaturation and elongation of the essential fatty acids. Furthermore, these changes could take place during the sperm maturation process.

## Conclusion

The composition of LC-PUFAs in sperm was changed by the dietary supplementation of FA sources. Fish oil supplementation significantly increased the concentration of DHA in sperm lipids. Our experiment demonstrated that after the consumption of commercial diets, C16:0 and C18:1 are the most prominent FAs in ram sperm. Consumption of FA in ram diet, changes FAs profile and this change has a persistency up to 35 days after excluding of FO in ration.

Nutritionists and physiologists should pay attention to this crucial point. The use of PUFA in ruminant ration is very useful, in spite of the fact that biohydroganation can cause lots of changes in physico-chemical characteristic and function in FA.
